# Intrinsic disorder is an essential characteristic of components in the conserved circadian circuit

**DOI:** 10.1186/s12964-020-00658-y

**Published:** 2020-11-11

**Authors:** Jacqueline F. Pelham, Jay C. Dunlap, Jennifer M. Hurley

**Affiliations:** 1 Department of Biological Sciences, Rensselaer Polytechnic Institute, Troy, NY 12180 USA; 2 Department of Molecular and Systems Biology, Geisel School of Medicine at Dartmouth, Hanover, NH 03755 USA; 3 Center for Biotechnology and Interdisciplinary Sciences, Rensselaer Polytechnic Institute, Troy, NY 12018 USA

**Keywords:** Circadian clock, *Neurospora crassa*, FREQUENCY, PERIOD-2, Oscillator mechanism

## Abstract

**Introduction:**

The circadian circuit, a roughly 24 h molecular feedback loop, or clock, is conserved from bacteria to animals and allows for enhanced organismal survival by facilitating the anticipation of the day/night cycle. With circadian regulation reportedly impacting as high as 80% of protein coding genes in higher eukaryotes, the protein-based circadian clock broadly regulates physiology and behavior. Due to the extensive interconnection between the clock and other cellular systems, chronic disruption of these molecular rhythms leads to a decrease in organismal fitness as well as an increase of disease rates in humans. Importantly, recent research has demonstrated that proteins comprising the circadian clock network display a significant amount of intrinsic disorder.

**Main body:**

In this work, we focus on the extent of intrinsic disorder in the circadian clock and its potential mechanistic role in circadian timing. We highlight the conservation of disorder by quantifying the extent of computationally-predicted protein disorder in the core clock of the key eukaryotic circadian model organisms *Drosophila melanogaster, Neurospora crassa,* and *Mus musculus*. We further examine previously published work, as well as feature novel experimental evidence, demonstrating that the core negative arm circadian period drivers FREQUENCY (*Neurospora crassa*) and PERIOD-2 (PER2) (*Mus musculus*), possess biochemical characteristics of intrinsically disordered proteins. Finally, we discuss the potential contributions of the inherent biophysical principals of intrinsically disordered proteins that may explain the vital mechanistic roles they play in the clock to drive their broad evolutionary conservation in circadian timekeeping.

**Conclusion:**

The pervasive conservation of disorder amongst the clock in the crown eukaryotes suggests that disorder is essential for optimal circadian timing from fungi to animals, providing vital homeostatic cellular maintenance and coordinating organismal physiology across phylogenetic kingdoms.

Video abstract

**Graphical abstract:**

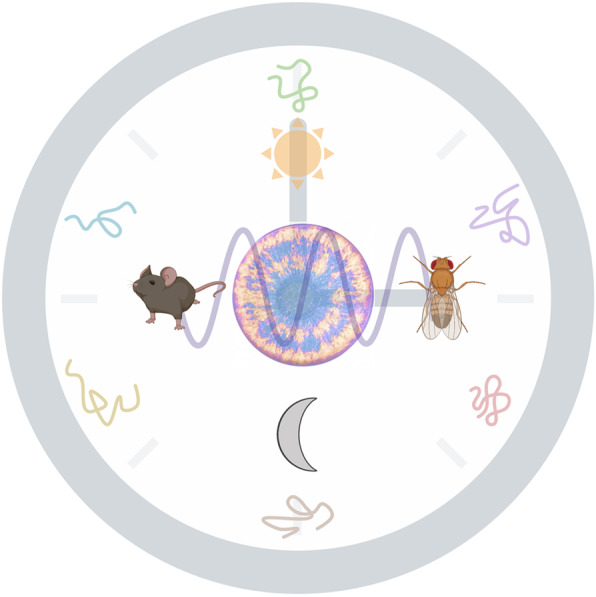

## Background

Over the last several billion years, only a select number of environmental stimuli have remained constant on planet Earth, one being the Earth’s diurnal cycle. The dependability of this cycle has facilitated the evolution of anticipatory time-keeping mechanisms across all kingdoms of life. These predictive biological phenomena confer an evolutionary advantage, increasing both survival and reproductive rates in the organisms in which they evolved [1–4]. The reach of these rhythms is vast, spanning many facets of biology with almost all mesophilic cells maintaining a circadian clock. The regulation of everything from gene expression to cellular homeostasis depends on these circadian rhythms, from unicellular prokaryotes needing to time energy production with the rising of the sun, to complex higher eukaryotes with multiple organs and tissue types needing synchronicity (reviewed in [5]). For example, the elegant heliotropism of the sunflower is a circadian behavior that enables its pre-sunrise redirection. This circadian timing increases the surface temperature of the flower and thus the number of pollinators attracted to its surface as compared to sunflowers that do not redirect their flower to anticipate sunrise [4].

**Fig. 1:**
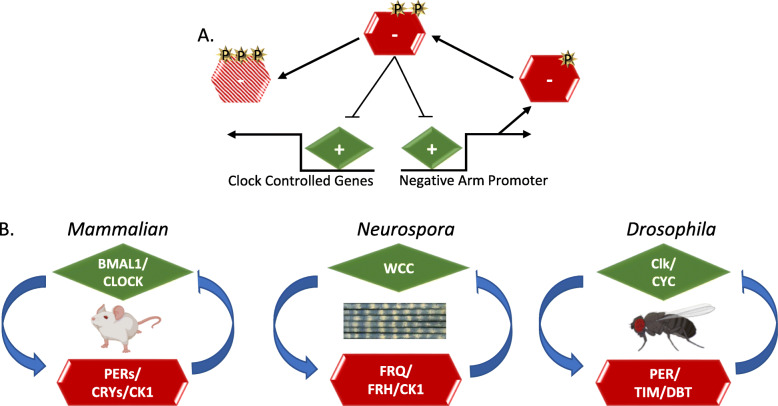
Conservation of the Core Clock Architecture. **a** The Transcription-Translation Negative Feedback Loop (TTFL) in fungi and animals comprises two main complexes. The first is a pair of heterodimeric activators known as the positive arm complex (green diamond) and the second, a repressing negative arm complex (red hexagon). The positive arm drives the expression of genes encoding negative arm components leading to the transcription and translation of the negative arm proteins. The negative arm proteins complex with kinases and are post-translationally modified by phosphorylation (shown as yellow stars), enabling negative arm repression of the positive arm. Distinct phospho-states trigger both positive-arm repression (blunted arrow) and negative-arm protein instability (represented by the faded red hexagon). Phosphorylation-related turnover is not necessary to close the loop in fungi, where this has been examined most closely, but instead represents cellular good housekeeping, cleaning up proteins that are no longer useful. When active, the positive arm also promotes the expression of clock-controlled genes (*ccg*s), which are predicted to control circadian output (reviewed [15]) **b** In the mammalian clock, positive arm-proteins Brain and Muscle ARNT-Like 1 (BMAL1, also known as ARNTL) and Circadian Locomotor Output Cycles Kaput (CLOCK) form a complex to activate the negative-arm PERIODs (PER1, PER2, and PER3) in addition to CRYPTOCHROMEs (CRY1 and CRY2). PERs and CRYs complex with several kinases to repress CLOCK:BMAL activity [16]. In the *Neurospora* clock*,* the positive heterodimer complex is comprised of White Collar-1 (WC-1) and White Collar-2 (WC-2) which together form the White Collar Complex (WCC). WCC drives the expression of the negative arm component FREQUENCY (FRQ), which, with its essential binding partner FREQUENCY-Interacting RNA Helicase (FRH), associates with the kinase Casein Kinase-1 (CK-1a) forming the FRQ-FRH Complex (FFC) [23, 24]. The positive-arm constituents in the *Drosophila* clock are CYCLE (CYC) and dClk, while the negative arm components are PERIOD (dPER), TIMELESS (TIM) and casein kinase 1, called DOUBLETIME (DBT) in Drosophila [25]. Figure 1b was created using BioRender.com

These overt circadian behaviors have been observed for hundreds of years, with the first true circadian oscillation recorded by the astronomer de Marian in the leaf movements of a Mimosa plant in 1729 [6, 7]. Since de Marian’s initial observation, research in the field of circadian rhythms has progressed significantly, including the characterization of the mechanisms that time rhythms at the molecular level in several model organisms, which led to the award of the Nobel prize in medicine and physiology in 2017 [8–12]. The central molecular oscillator that coordinates these rhythms is referred to as the “core circadian clock”. In animals and fungi, this clock comprises a protein orchestrated transcription-translation negative-feedback loop (TTFL), with the positive arm stimulating the expression of the negative arm [13, 14]. The negative arm then inactivates the positive arm and represses its own creation, completing its tightly-timed lifecycle before being inactivated by multiple phosphorylations. This allows the positive arm to reinitiate the cycle, restarting the clock (Fig. 1a) [15, 16]. The regulation of genes beyond those of the negative arm of the clock by the positive arm creates the bridge between the core timekeeper and circadian behavior. The number of genes under the control of the clock varies by organism and tissue type but is thought to be extensive, with as much as 80% of mammalian genes predicted to be under circadian transcriptional regulation across all mammalian tissue types [17–20]. This transcriptional regulation leads to an extensive amount of physiological fluctuations over the circadian day [17, 21, 22].

With this sizeable amount of gene regulation, and by extension physiology, it is no surprise that the disruption of circadian rhythms has been noted to lead to decreased physiological fitness [3, 26]. Chronic circadian disruption has been linked with many disease states, including a higher risk of obesity, heart disease, diabetes, depression, and cancer [27–31]. Furthermore, genes under the control of the circadian clock show a strong correlation with disease-associated genes and out of the top 100 selling drugs in the US, 56 target a gene under circadian regulation (including the top 7) [32]. With 20% of the working population subject to “shift work” and the widespread use of digital screens and blue-light-emitting devices, circadian disruption is a growing cause of preventable disease [33, 34].

Conversely, methods of circadian intervention and treatment have been developed, including the application of light-based therapy for dementia-related pathologies. For example, Alzheimer’s disease commonly results in disturbances to circadian rhythms, leading to increased deposition of the plaques that exacerbate Alzheimer’s disease pathology. This in turn leads to further dysregulation of the circadian clock, thus creating a positive feedback loop with negative effects on the pathology and progression of Alzheimer’s disease. As a treatment modality, optimized circadian entrainment lighting for patients with Alzheimer’s disease has been implemented in assisted living facilities and has been shown to improve behavior, sleep, and mood [35]. In summary, the circadian system has been shown to play an essential role in organismal health and physiology, though many questions still remain detailing its extensive reaches into the cell.

### Circadian output and conserved molecular oscillators

The general principles that govern the TTFL that orchestrates circadian rhythms are conserved in most eukaryotic species, and fungal, insect, and mammalian clocks all have some variation of the same basic clock architecture (Fig. 1) [15, 23]. In the case of fungal and animal clocks, the positive arm of the clocks consists of a pair of heterodimeric transcription factors that drive the translation of the negative arm proteins [36–38]. These negative arm complexes generally consist of several proteins, including a core time-keeping protein(s), stabilizers for that core protein(s), and kinases, which temporally phosphorylate the core time-keeping protein(s) (Fig. 1b). A conserved feature of the protein complexes of the clock lies in their distinctive interaction mechanism. Uniquely, all clock proteins form heterodimers that interact via a PAS domain [37, 39]. Phosphorylation of the clock time-keeping protein(s) determines both the core time-keeping proteins’ repressive activities on the positive arm, the half-life of the negative arm, and the transcriptional activity of the positive-arm proteins [9, 15, 16, 40]. The negative arm complex coalesces in the cytoplasm before it migrates to the nucleus to repress positive-arm activity, after which it is ubiquitinated and targeted for degradation.

This transcriptional activity of positive arm/transcription factor complexes extends beyond the promoter of the core time-keeping protein and also regulates a host of other promoters in the cell. The rhythmic transcriptional activation on the promoters of these genes from the positive arm is believed to be the primary mechanism of output. In addition, recent work in several eukaryotic species has also shown that there is a great deal of post-transcriptional regulation, for example rhythmic proteins arising from non-rhythmic transcripts [22, 41–45]. This suggests that the canonical TTFL control of output has several as yet undiscovered levels of regulation.

**Fig. 2:**
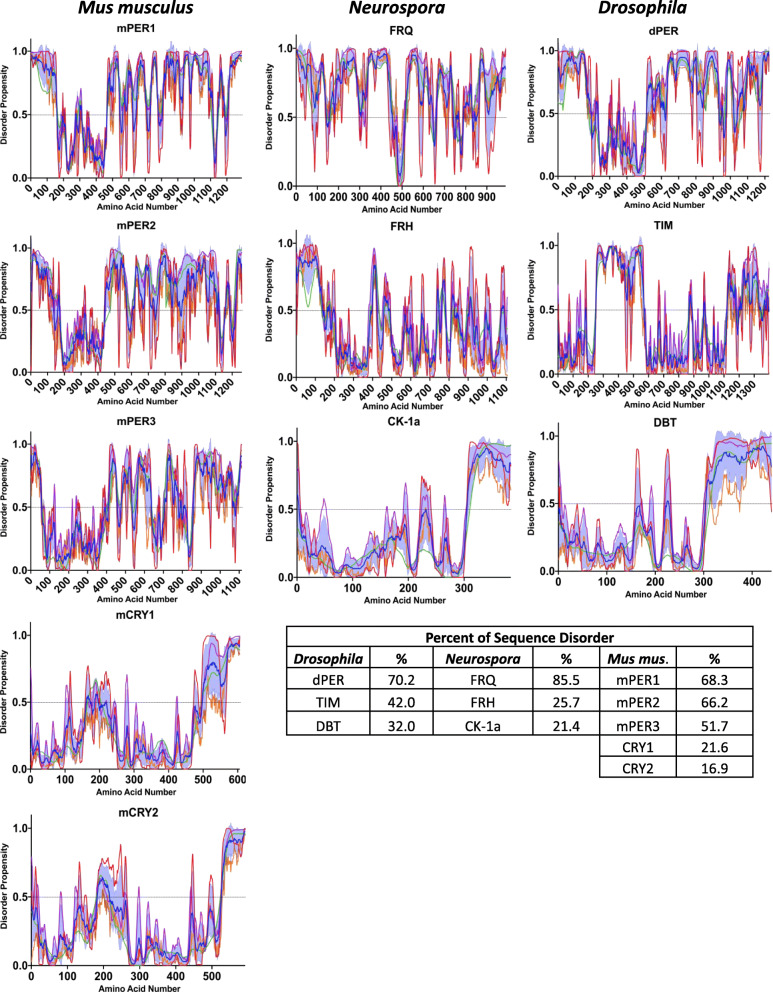
Computational Analysis Demonstrates Disorder in the Negative-Arm Constituents. Plots of the disordered propensity scores vs the primary amino acid sequence of the negative-arm components from *Murine*, *Neurospora*, and *Drosophila* clocks. The propensity scores were calculated using four algorithms, the first three from the PONDR family VLXT (red line), VL3-BA (green line) and VSL2 (purple line) and the last from IUPred2A long (orange line). The mean is plotted as the blue line with the SD distribution shaded in light blue [51–54]. Any residue scoring 0.5 or higher in the mean calculation was considered toward the percent disorder calculation

While the conserved architecture of the TTFL has allowed for significant investigations and discoveries to be inferred across multiple circadian systems and the basic molecular mechanism of the TTFL is appreciated, many of the complex biophysical details that underlie how these oscillators operate have yet to be determined. A good example of this lies in the lack of sequence conservation of the negative arm of the circadian clock, which is one of the reasons it has been difficult to discern the mechanism of action of the negative arm proteins [46, 47]. However, while there is little sequence conservation, one commonality in the negative arm proteins of eukaryotic TTFLs is in the conservation of intrinsic disorder in negative arm proteins [48–50] (Fig. 2).

Intrinsically Disordered Proteins (IDPs) and Intrinsically Disordered Protein Regions (IDPRs) are amino acid sequences that lack a fixed, three-dimensional, structure. These IDPs and IDPRs exist instead in a heterogenous ensemble of conformations and can be found ubiquitously in macromolecular complexes [55]. In fact, protein disorder is known to play a role in many facets of biology across the kingdoms of life and their conformational plasticity confers a level of stochasticity and tuneability to many biological systems, the import of which we are only now beginning to appreciate [56]. In the following discussion of intrinsic disorder in clock proteins, to delineate the difference between an IDP and an IDPR, we have chosen to use the IDP/IDPR classification method suggested by Deiana et al. 2019 with slightly modified naming [57]. To quantify the level of disorder, four algorithms were used and averaged together. The first three predictions are from the PONDR family (VLXT, VL3-BA and VSL2) and the last is from IUPred2A (long) [51–54, 58]. We classify proteins that have more than 30% of their residues predicted to be disordered as IDPs. We classify proteins that have less than 30% of their residues predicted to be disordered but have either a C or N-terminal disordered segment longer than 30 consecutive residues, or a segment longer than 40 consecutive disordered residues in positions distinct from the N and C-terminus, as proteins with IDPRs. Ordered proteins (ORDPs) are classified as having less than 30% predicted disordered residues and no disorder predicted in their C or N-terminal segments that are longer than 30 residues or non-terminal disordered regions longer than 40 residues.

### *Neurospora crassa* as a circadian model organism

Model systems have long been a tenet of biological investigation, allowing for the distillation of complexities for a greater understanding of the problem at hand. Due to the fact that the propensities of evolution have converged on efficiency mechanisms for timekeeping, we have, and can still, learn much about the clock from tractable model systems. One such circadian model, *Neurospora crassa,* a filamentous fungus, has been a vital model organism across many disciplines of biology [59–61]. It originally gained notoriety as the model organism used by Beadle and Tatum in their Nobel prize winning mutant screens that yielded their one-gene-one enzyme hypothesis. With both sexual and asexual reproductive cycles and a fully sequenced genome, *Neurospora* has continued to be an efficient system for use in genetic and molecular research in many subdisciplines [62, 63]. In circadian biology, *Neurospora* has been used for over half a century [11, 23, 64, 65]. The investigation for a molecular explanation of a cellular oscillator began in *Neurospora* and much of what has been learned about animal clocks was first discovered by studying *Neurospora.* This includes many of the biochemical activities assigned to clock proteins and the first experimental evidence of negative feedback oscillations, which was demonstrated with the *frequency* gene (reviewed in [49]). *Neurospora* continues to serve as an efficient model organism for circadian characterization from the molecular to “omics” scale [22, 48, 66–68].

Notably, *Neurospora* was the first organism in which IDPs were demonstrated to play a role in the clock. Furthermore, *Neurospora* makes extensive use of disordered proteins and disordered regions as clock components (Figs. 2 and 4). In fact, *Neurospora* possesses one of the most disordered proteomes of all eukaryotic clock model organisms. Intrinsic protein disorder is a proteome wide phenomenon across all kingdoms of life and has been suggested as an evolutionary mechanism of complexity [69, 70]. When enumerating long disordered regions of 30 residues or longer, it was found that 23.9% of the *Neurospora* proteome is classified as “long disorder”. Compared to other clock model organisms, including *Homo sapiens* (18.6% proteome-wide disorder), *Mus musculus* (17.4% proteome-wide disorder), *Drosophila melanogaster* (19.8% proteome-wide disorder), and *Arabidopsis thaliana* (11.5% proteome-wide disorder), *Neurospora* has significantly more protein disorder [71]. This increased proportion of disorder is not specific to the fungal lineage, as *Saccharomyces cerevisiae* demonstrates 14.6% proteome-wide disorder [71]. As disorder is more prominent in the *Neurospora* proteome, disorder is likely to play an enhanced role in the fungal clock, which possibly led to the early recognition of extensive protein disorder in the clock in *Neurospora*.

### Conformational disorder in the negative arm of the *Neurospora* clock

Residing at the heart of the circadian clock in *Neurospora* is the negative-arm protein FREQUENCY (FRQ) (Fig. 1b and 2). FRQ and its interactors are the principal drivers of the circadian period and, with the exception of a few small regions, is predicted to be largely intrinsically disordered [48–50, 72], with 85.5% of its sequence computationally presenting as disordered (Fig. 2) [54]. Further, FRQ was the first protein to be biochemically verified to be an IDP within any core clock [49, 50]. FRQ remains in solution after heat treatment while its partner and nanny protein, the highly ordered FRQ-interacting RNA Helicase (FRH), precipitates out of solution [50]. This is important as heat-stability is widely defined as a key indicator of IDPs as they are predicted to remain in solution after heat treatment while globular proteins unfold, then misfold upon cooling, and precipitate [73–77]. Another critical element supporting the IDP nature of FRQ is the fact that IDPs including FRQ commonly have “nanny” proteins that support and protect them [50, 78]. Moreover, FRQ is more rapidly degraded by treatment with proteinase K and Thermolysin than FRH, another indication of an IDP nature, as tightly folded proteins are able to inhibit protease cleavage by the protection of protease target sites [48, 50]. Finally, an isoform of FRQ without the first one hundred amino acids of the protein, short-FRQ, was surveyed with Circular Dichroism (CD) and demonstrated to have a mostly unstructured spectra, with only a slight dip in the helical range, most likely due to the only, small, predicted region of structure on FRQ, a coiled coil-domain [49, 50, 79]. Though the role of FRQ as a core clock constituent was defined over 25 years ago, much is lacking in the understanding of the molecular function of FRQ in the circadian system as standard structural, mechanistic, and expression paradigms have failed, presumably due to the significant level of disorder in FRQ [48, 67].

IDP conformational regulation is heavily influenced by phosphorylation, and conformational modulation via post-translational modifications can facilitate the tunability of both protein function and protein stability, which has been detailed in many other systems [56, 80–83]. Importantly, phosphorylation is an essential component of circadian timing and circadian protein stability in the *Neurospora* clock [50, 72, 80, 81, 83, 84]. FRQ is extensively phosphorylated, over 100 times throughout the course of the circadian day [84, 85]. Numerous FRQ phosphomutants have been tested, yielding both lengthened and shortened clock period phenotypes, reiterating the importance of phosphorylation on clock regulation [11, 38, 84]. Interestingly, the patterning of FRQ phosphorylation appears to cluster around regions of disorder and the extent of FRQ temporal phosphorylation could impose bulk electrostatic effects [48, 81]. Moreover, this corresponds with the increased coincidence of post-translational modifications occurring within disordered regions and the idea that intrinsic disorder is important for phosphorylation [86, 87]. Recent investigations into the IDP nature of FRQ have demonstrated temporal conformational differences through limited digestion, conformational changes which are suggested to be induced by a repulsion of the N and C-terminus induced by phosphorylation, exposing PEST regions that may target FRQ for degradation [48, 72, 88]. Considering the asymmetric charge distribution of FRQ, coupled with flexibility facilitated by the high degree of disorder, this suggests that phosphorylation may be the primary driver that allows FRQ to exhibit a high degree of dynamic and temporal conformational plasticity [72].

Phosphorylation is also known to play a role in transmitting signals between distinct regions in conformationally flexible proteins to facilitate the potential for molecular allostery to enhance interactions with biological partners [81, 89–91]. Moreover, the absence of structure in a protein with numerous binding partners provides a thermodynamic advantage, allowing specificity and affinity to be tuned for many partners [83, 92, 93]. One mechanism thought to facilitate the “one-to-many” binding hypothesis are molecular recognition features (MoRFs), short disordered amino acid sequences 10–70 residues in length which act as the initial molecular recognition step in binding [94]. MoRFs are suspected to undergo a disorder to order transition upon binding and serve critical regulatory roles for interactions in signal transduction and cell regulation [87]. PTMs are known to occur in MoRF regions, which could suggest a tunable and context dependent mechanism for binding and regulation of these regions [87, 95].

Considering the combinatorial effect of the 100 phosophosites identified on FRQ, the number of FRQ-phosphostates reaches the realm of 2^100^, facilitating the potential for an extensive level of regulation, in addition to the ascribed static timekeeping function of FRQ. Recognizing that FRQ and other disordered negative-arm proteins in eukaryotic clocks act as the interaction hub for many clock proteins, allosteric effects due to phosphorylations could impart a level of circadian regulation via the temporal timing of circadian-interacting partners, which would confer a fine-tuning method to the circadian protein-protein interaction network [84, 96]. Importantly, this phosphorylation/conformation paradigm is mirrored in higher eukaryotes [97]. Extrapolating from FRQ and the *Neurospora* clock, this suggests that phosphorylation of IDPs is a primary timing mechanism for core circadian clock protein-protein interactions [98].

FRQ is expressed as two isoforms, a long form (L-FRQ, a.a. 1–989) and a short form (S-FRQ, aa 100–989) which is missing the first 99 residues [99]. Both isoforms generate robust rhythms but the ratio of expressed isoforms varies as a result of temperature mediated alternative splicing and impacts the clock’s temperature response [100]. At lower temperatures, there is a decrease in the amount of L-FRQ. As temperatures rise, the ratio skews in favor of L-FRQ, with very low amounts of S-FRQ remaining at the 30 °C range [101]. An emerging theme in the regulation and behavior of IDPs and IDRs is the correlation and cooperativity of disorder with alternative splicing and post translational modifications, known as the IDP-AS-PTM tool kit for signaling diversification [102–105]. With numerous examples across the eukaryotic kingdom, it has been suggested that this tool kit allows for versatility and the ability to accomplish dynamic regulatory function with the capacity to respond to changing environments [105]. The disordered nature of the first 100 aa of FRQ, its context dependent splicing and expression, and the approximate 15 phosphosites in this region, suggests that circadian regulation in the N-terminus of L-FRQ follows the IDP-AS-PTM paradigm. Moreover, the synergism of the IDP-AS-PTM tool kit is conserved in the clocks of higher eukaryotes [105].

Disorder in the *Neurospora* clock exists beyond FRQ. Once the positive arm triggers the transcription and translation of FRQ, FRQ dimerizes and immediately binds with its chaperone/nanny protein FRH, forming the FRQ-FRH Complex (FFC) [50, 106, 107]. FRH is an orthologue of the yeast TRAMP complex member Mtr4 [50]. Mtr4 activates the nuclear RNA exosome and regulates RNA metabolism with a preference for poly-adenylated mRNA [108, 109]. FRH is essential for both survival and clock functionality in *Neurospora*, though these functions are not synonymous [50, 106]. Binding between FRQ and FRH is imperative for FRQ localization, proper phosphorylation, and stability [50, 107, 110]. Though FRH has been structurally characterized to be highly ordered, it has a significantly disordered N-terminus (169 residues) with 25.7% of its entire sequence predicted to be disordered (Fig. 2) [50, 108]. This disordered N-terminal region is key to the binding between FRQ and FRH and deletions within this region cause arrhythmicity [50]. Binding with FRQ was demonstrated to occur between amino acids 100–150 in FRH, and mutants within this region cause a clock period change as well as a change in affinity for the positive arm of the *Neurospora* clock, the White-Collar Complex (WCC) [50, 111]. Of note, the FRH^V142G^ mutant showed the greatest change to the complex and clock period, weakening the interaction between FRH, FRQ and the WCC, and yielding a 18.7 h period (~ 21.5 h WT) [111]. Relevantly, this disordered N-terminal region in FRH is not present in Mtr4 or other orthologues from fungal species that do not maintain an orthologue of FRQ, suggesting it evolved specifically to function in circadian regulation. IDP/IDPR interactions are common, often driven by opposing charge distributions, and do not necessarily lead to a loss of conformational heterogeneity in the binding partners [112–115]. Considering the positive charge distribution of FRQ and the negative charge of the disordered FRH N-terminus, this is a tantalizing mechanism to consider for the modulation of the interaction between the negative arm *Neurospora* clock proteins.

### Conformational disorder in the negative arm in other eukaryotic clocks

**Fig. 3:**
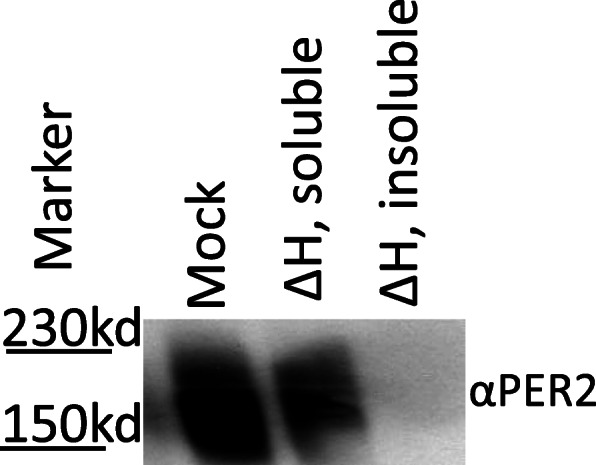
Heat treatment of mPER2 demonstrates that mPER2 has the biochemical characteristics of an IDP. To demonstrate that mPER2 remained soluble after heat stability, murine liver lysates were subjected to heat treatment (∆H: 100 °C for 10 min) with a mock treatment on ice in parallel. Centrifugation was used to separate the soluble fraction from the aggregated proteins. For western blot visualization, 10 μg of total protein was loaded per lane for all treatments, with the Santa Cruz Per-2 H-90 (sc-25,363) antibody used for detection

IDPs are conserved in the negative arm beyond the *Neurospora* clock. In mammals, as well as in *Drosophila*, the core negative arm proteins are the PERIODs (PERs) and these proteins are functional analogs of FRQ (Fig. 1b) [50, 83]. As with FRQ, computational analysis of disorder in the PERs, including human (hPER1, 2, and 3), murine (mPER1, 2, and 3), and *Drosophila* (dPER), indicates that the PERs are IDPs, with mPER2 and dPER at 66.2 and 70.2% predicted sequence disorder respectively (Fig. 2) [48, 83]. Furthermore, like its counterpart FRQ, we have shown that mPER2 extracted from murine liver cells remains in solution after heat treatment (Fig. 3) using the same approach as we used on FRQ. This demonstrates that mPER2 also displays some of the classical biochemical characteristics of an IDP [73–77].

In parallel with the IDP conservation between FRQ and the PERs, dPER and mPER have been shown to be highly temporally phosphorylated and the temporal phosphorylation of the PERs is known to serve many regulatory roles, including localization, transcriptional repressor potency, temperature compensation, and protein stability [83, 97, 116]. Limited proteolysis of dPER also demonstrated that hyperphosphorylated dPER has a more open conformation, potentially allowing it to be more readily accessible for targeted degradation [117]. It has also been suggested that mPER2, like FRQ, may be supported by a nanny helicase [50]. mPER2 complexes with two DEXD-box helicases, DDX5 (DEAD-box protein 5) and DHX9 (DEAH-box protein 9). Deletion of either results in a shortened period and increased rates of mPER2 transcription, both predictable outcomes if these DEXD-box helicases played a role in PER stability as FRH does for FRQ [50, 118]. Along these lines, mPER2 and hPER2 have been demonstrated to have a multitude of interacting partners that serve to regulate clock function [96, 118, 119]. It is known that a high level of protein-interaction promiscuity is a common feature for IDPs, as they often serve as hub nodes in protein interaction networks [120–124]. Considering the conservation of the aforementioned characteristics between FRQ and PER, this bolsters the idea of a critical need for intrinsic disorder in clock proteins such that they can serve as flexible interaction hubs.

Beyond PER, higher eukaryotes have several additional components that serve critical roles in the negative arm complex that also have large regions of intrinsic disorder, including Cryptochrome 1 (CRY1) [83, 125–127]. Computationally, mCRY1 is an IDPR displaying 21.6% sequence disorder (Fig. 2). CRY1 contains a significantly disordered C-terminal tail (~ 100 aa in hCRY1 and ~ 200aa in *A. thaliana* CRY1) (Fig. 2) [126]. The mCRY1 C-terminal tail was further defined as disordered via CD and Analytical Ultracentrifugation (AUC) [128]. Modification or truncation of the mCRY1 tail alters the period as well as the amplitude of the rhythms [129–133]. As with disordered regions in FRQ and PER, the C-terminal disordered tail of CRY1 is temporally phosphorylated (8 confirmed phosphosites in the tail region) and is critical for regulating circadian period and amplitude [98, 130, 134]. The mCRY1 tail interacts with the N-terminal photolyase homology region (PHR) domain on mCRY1, and it is suggested that this auto-interaction is essential for proper circadian timing [127]. This binding is believed to be involved in a time-of-day specific competitive interaction between mCRY and either the CLOCK/BMAL1 complex or PER2 [127]. Interestingly, the binding between PER2 and CRY1 is via an intrinsically disordered CRY1-binding domain on PER2, mirroring the IDP/IDPR interaction of FRQ and FRH [50, 127].

Moreover, the C-terminal CRY1 tail is produced from the splicing of 3 exons, exons 10, 11, and 12. In humans, it has been shown that an adenine-to-cytosine transversion in exon 11 results in exon skipping, creating the hCry1 *∆* 11 allele. This hCry1 *∆* 11 allele results in the lengthening of the clock period, increased hCRY1 nuclear localization, and an enhanced interaction with higher eukaryote positive-arm clock proteins, hCLOCK and hBMAL1. The effects of this mutation in hCRY1 resulted in an established case of human delayed phase sleep disorder, demonstrating the physiological importance in the proper functioning of the disordered regions of clock proteins [129].

The IDP-AS-PTM tool kit also extends to the negative arm of higher eukaryotic clocks. The negative arm of *D. melanogaster* is predicted to be disordered, with dPER and TIM at 70.2 and 42% respectively (Figs. 1 and 2). Alternative splicing results in the expression of 7 isoforms of *tim*. At the protein level, TIM has been found expressed in 4 of those isoforms and the isoform ratios are environmentally dependent and, like *Neurospora*, are suspected to play a role in the temperature response of the clock [135]. Furthermore, TIM and dPER heterodimerize and repress their expression in the nucleus [136–141]. Finally, the temporal phosphorylation of dPER and TIM, which is essential for regulating period length, fulfills the remaining qualification of the IDP-AS-PTM in the *Drosophila* clock [137, 142–144]. Moreover, splicing machinery and numerous kinases and other post-translationally modifying enzymes are under circadian regulation in *N. crassa, D. melanogaster* and *M. musculus* [19, 145–147]*.* The circadian timing of these processes likely extends circadian regulation using the AS-IDP-PTM tool kit to a meta scale in the cell.

### Conformational disorder in the positive arm of the clock

**Fig. 4:**
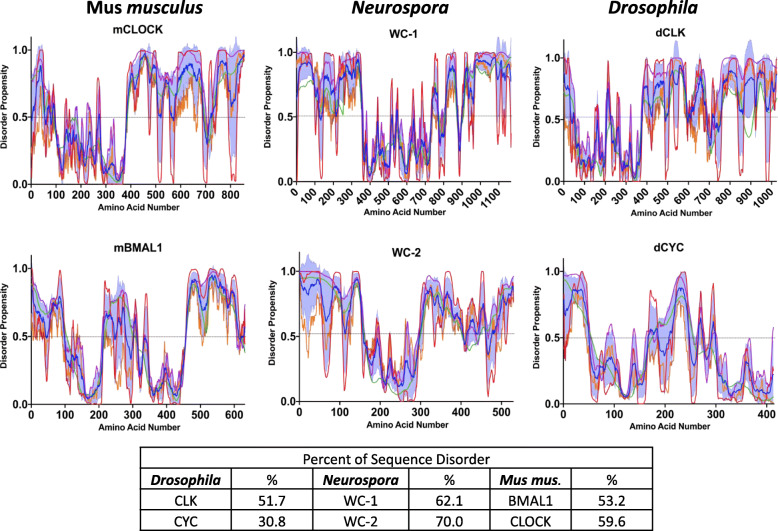
Computational Analysis Demonstrates Disorder in the Positive-Arm Constituents. Plots of the disordered propensity scores vs the primary amino acid sequence of the negative-arm components from *murine*, *Neurospora*, and *Drosophila* clocks. The propensity scores were calculated using four algorithms, the first three from the PONDR family VLXT (red line), VL3-BA (green line) and VSL2 (purple line) and the last from IUPred2A long (orange line). The mean is plotted as the blue line with the SD distribution shaded in light blue. Any residue scoring 0.5 or higher in the mean calculation was considered toward the percent disorder calculation

In addition to the disorder in the negative arm, the positive arm proteins of fungi and animals, which do share some sequence conservation, also have significant conservation of protein disorder (Fig. 4) [47]. As evidence is emerging that the majority of eukaryotic transcription factors are IDPs, with nearly two thirds of their sequence predicted to be disordered, it is logical the importance of disorder extends to the transcriptional activators of the positive arm of the clock [69, 103, 148–152].

In *Neurospora*, the heterodimer complex of the positive arm is comprised of White Collar-1 (WC-1) and White Collar-2 (WC-2), together known as the White Collar Complex (WCC) (Fig. 1b) [153]. Computationally, WC-1 and WC-2 are considered IDPs, with 62.1 and 70% of their respective sequences computationally classified as disordered (Fig. 4). Like FRQ, the WCC is highly phosphorylated, with 80 phosphosites mapped on WC-1 and 15 phosphosites mapped on WC-2 [66]. Also similar to FRQ, the phosphosites cluster in regions of disorder and these regional clusters of phosphosites play a role in the circadian feed-back loop, implying that conformational flexibility and plasticity is important for rhythmic core clock activity and output in both the positive and negative arms [66].

FRQ interacts with the WCC and appears to recruit kinases to impart WCC phosphorylation to repress WCC transcriptional activity, which is critical for proper circadian timing [66, 84, 154]. The interaction between FRQ and the WCC occurs within the Defective in Binding DNA domain on WC-1 (DBD) [155]. This region, albeit small, is also disordered, demonstrating a further point of evidence for disorder regulating the interaction of the core proteins of the clock and further supporting the role of intrinsic disorder in circadian regulation.

In higher eukaryotes, the constituents of the positive arm complex include the transcription factors CLOCK and BMAL1. Several regions of the proteins in this complex have been demonstrated to be disordered and computational analysis shows overall that mBMAL1 is predicted to be 53.2% intrinsically disordered while mCLOCK is predicted to be 59.6% intrinsically disordered (Fig. 4) [128]. As in lower eukaryotes, disorder has been characterized as an essential element in the interaction between the positive and negative arm proteins of the clock, and changes in these disordered regions play a role in the regulation of BMAL1 and CLOCK activity [83, 156–161]. More specifically, the C-terminal trans-activation domain (TAD) on BMAL1 was demonstrated by chemical shift dispersion to be an intrinsically disordered region and CRY1 competes for binding on this TAD region, binding which serves as a functional switch to activate/inactivate CLOCK/BMAL transcriptional activity [125]. “Locking” this Trp-Pro isomerization switch in the TAD of BMAL1 lengthens the circadian period, highlighting how conformational dynamics play a key role in circadian period determination, parallel to what has been suggested in *Neurospora* [48, 72, 125, 162].

The occurrence of low complexity prion-like domains (PrLDs), specifically Poly-Q regions, in the positive arm clock proteins from lower to higher eukaryotes [163–165], including *Arabidopsis* [166, 167], have been shown to play a role in period and phase regulation, and have an undefined mechanism. Considering the fact that Poly-Q regions are known to play a role in phase separation [168] at the transcript level, it has not escaped our notice that biomolecular condensation or phase separating behavior may play a role in the regulation of the clock. This could provide the capacity for numerous regulatory roles; from pre-transcription and the coordination of gene expression, to post-transcriptional regulation. Phase separation underscored by low complexity prion like domains (poly-Qs) have recently been demonstrated as a mechanism of transcriptional factor regime control in the fungi *C. albicans* in its phenotypic pathogenicity switch process [169]. Interestingly, another phase separation phenomena, the formation of P-bodies, has been demonstrated to be under circadian clock control [170]. P-bodies, or processing bodies, are widely conserved foci within the cell that are formed by phase separation [171]. These biomolecular condensates house many enzymes that are involved with mRNA turnover, including DDX proteins, and have also been demonstrated to store mRNA prior to translation. Of note, over half of the proteins identified in a screen of the components in P-bodies are DDX proteins and their interacting partners [172] making the this, and aforementioned ideas, alluring avenues for the explanation of potential mechanisms of circadian pre and post-transcriptional regulation.

## Conclusions

IDPs have been suggested to serve important roles in finely tuning the circadian regulatory circuit through post-transcriptional regulation, multiple interacting partners, and many other mechanisms. We have discussed examples of disorder in the clock and how they underlie the mechanisms for several of the phenomena in the circadian system from timing, to the regulation of output, to providing the capacity for the clock to respond to cellular conditions. Considering the complexity and deep reaches of the molecular circadian circuit, disorder presents an essential and inherent mechanism of biological control, supported by the conservation of protein disorder in clocks from lower to higher eukaryotes.

## Data Availability

Protein sequences for analysis were acquired from UniProt or FungiDB.

## Abbreviations

AUC: Analytical Ultracentrifugation
BMAL: Brain and Muscle ARNT-Like 1
CD: Circular Dichroism
CLOCK: Circadian Locomotor output cycles kaput
CRY1: Cryptochrome 1
DBD: Defective in Binding DNA Domain
DDX5: DEAD-box protein 5
dPER: *Drosophila* PERIOD
DDX9: DEAD-box protein 9
FFC: FRQ-FRH Complex
FRH: FREQUENCY-Interacting RNA Helicase
FRQ: FREQUENCY
hCRY: human Cryptochrome 1
hPER: human PERIOD
IDP: intrinsically disordered protein
IDPRs: intrinsically disordered protein regions
mBMAL: murine Brain and Muscle ARNT-Like 1
mCRY1: murine Cryptochrome 1
mPER1: murine PERIOD 1
mPER2: murine PERIOD 2
mPER3: murine PERIOD 3
Mtr4: yeast mRNA Transport Mutant − 4
ORDP: ordered proteins
PAS: PER ARNT SIM
PER: PERIOD
PEST: Proline Glutamic Acid Serine Threonine sequence
PrLDs: Prion-like domains
TAD: trans-activation domain
TRAMP: Trf4/Air2/Mtr4p Polyadenylation complex
TTFL: transcription-translation negative-feedback loop
WC-1: White Collar-1
WC-2: White Collar-2
WCC: White Collar Complex

## Protein UniProt IDs used for disorder analysis

FRQ: P19970
FRH: Q1K502
WC-1: Q01371
WC-2: P78714
CK-1a: V5IQ40
mPER1: O35973
mPER2: O54943
mPER3: O70361
mCRY1: P97784
mCRY2: Q9R194
mBMAL1: Q9WTL8
mCLOCK: O08785
dPER: P07663
TIM: P49021
dCLOCK: O61735
DBT: O76324
dCYC: O61734
